# A Comparative Evaluation of the Apical Dimensional Variability of ISO Size 30 Gutta-Percha Cones With 0.04 and 0.06 Tapers From Two Different Manufacturers

**DOI:** 10.7759/cureus.87341

**Published:** 2025-07-05

**Authors:** Ceren Turan Gökduman, Esra Arılı-Öztürk

**Affiliations:** 1 Endodontics, Trakya University, Edirne, TUR

**Keywords:** diameter, gutta-percha, standardization, taper, variability

## Abstract

Introduction: Gutta-percha (GP) is the most commonly used core material in root canal obturation. This study aimed to evaluate the dimensional variability of ISO size 30 GP cones with 0.04 and 0.06 tapers from two different manufacturers: Ocean (Pearl Dent Co., Ltd., Ho Chi Minh City, Vietnam) and VDW.ROTATE (VDW GmbH, Munich, Germany).

Methods: ISO size 30 GP cones with 0.04 and 0.06 tapers were assigned to four groups based on the taper and manufacturer, with 60 GP cones in each group. The GP cone diameters were measured at 1 mm (D1), 2 mm (D2), and 3 mm (D3) from the tip using digital image analysis. Diameter increments between D1-D2 and D2-D3 were calculated to assess the influence of the manufacturer and taper on dimensional variability.

Results: No significant differences were found between manufacturers in the 0.04 taper group. In contrast, Ocean GP cones in the 0.06 taper group had significantly larger diameters at D1, D2, and D3 compared to VDW.ROTATE GP cones (p < 0.001, p < 0.001, and p = 0.005, respectively). VDW.ROTATE showed greater D2-D1 increases than Ocean at both tapers (p = 0.005 and p < 0.001) and a greater D3-D2 increase at the 0.06 taper (p = 0.005).

Conclusion: It was found that there was significant dimensional variability in gutta-percha cones resulting from manufacturing differences and taper variations in ISO size 30 cones. These inconsistencies can adversely affect apical sealing and treatment outcomes, emphasizing the need to consider them when choosing GP cones for clinical use.

## Introduction

Achieving three-dimensional, void-free root canal filling is essential to prevent bacterial contamination of the root canal system and periapical tissues [1]. The quality of root canal fillings has been reported to be a predictor of endodontic treatment outcomes [2]. Numerous studies have demonstrated that adequate filling length and density significantly increase the treatment success rate [3-5]. Gutta-percha (GP) is the most commonly used core material in root canal obturation due to its biocompatibility, ease of handling, and favorable physical and mechanical properties [6]. For successful apical sealing, GP needs to be well adapted to the canal walls both apically and laterally [1].

In root canal obturation, especially when using the single-cone technique, the compatibility between the apical diameter and taper of the GP cone and those of the NiTi files used for mechanical shaping is crucial for achieving a hermetic seal in the apical third [7]. For this reason, GP cones specifically designed to match the apical size and taper of the corresponding NiTi files are frequently manufactured [7]. However, discrepancies in cone tip sizes or mismatches between the taper of the GP cone and the canal may prevent the cone from reaching the working length, resulting in a thick layer of the sealer, which may also contribute to the reduced success of root canal treatment [8].

According to ISO (International Organization for Standardization) 6877, the tolerance of GP cones is ±0.05 mm for sizes 10 to 25 and ±0.07 mm for sizes 30 to 140 [9]. Therefore, nominal diameter and taper values reported by manufacturers may vary despite technically complying with current “standards” [10]. Several studies have emphasized the lack of standardization between endodontic instruments and GP cones [6,11,12], whereas others have focused on the dimensional inconsistencies of GP cones [10,13,14].

The aim of this study was to evaluate the dimensional variability of ISO size 30 GP cones with 0.04 and 0.06 tapers from two different manufacturers: Ocean (Pearl Dent Co., Ltd., Ho Chi Minh City, Vietnam) and VDW.ROTATE (VDW GmbH, Munich, Germany) using digital image analysis. The GP cone diameters were measured at three apical levels: 1 mm (D1), 2 mm (D2), and 3 mm (D3) from the tip. Diameter increments between D1-D2 and D2-D3 were calculated to assess the influence of the manufacturer and taper on dimensional variability.

## Materials and methods

Sample size

In accordance with a previous study [11], and based on a power analysis conducted with G*Power software (Heinrich-Heine University, Düsseldorf, Germany), with an alpha level of 0.05, power of 80%, and effect size 0.52, the estimated sample size required for each group was 60 (n = 60).

This in vitro experimental study was conducted in the laboratory of the Department of Endodontics at Trakya University Faculty of Dentistry (Edirne, Turkey) in April 2025. To ensure standardization, all photographs were taken at the same time of day, and measurements were performed at the same time of day. A single, pre-calibrated operator performed all measurements.

GP cones with 0.04 and 0.06 tapers from two different manufacturers (Ocean and VDW.ROTATE) were included in the study. To minimize the potential impact of manufacturing inconsistencies or dimensional alterations due to transportation and storage, GP cone samples were randomly obtained from various packaging and production lots.

The GP cones were kept under 23 ± 2 °C and 50%-65% relative humidity for 24 hours before testing. A total of 240 GP cones were individually affixed to millimeter paper, and images were captured using a Nikon camera (Model D7200; Nikon, Tokyo, Japan) mounted on a tripod and equipped with a 105-mm macro lens (Nikon AF-S VR Micro-NIKKOR 105mm f/2.8G IF-ED; Nikon).

**Figure 1:**
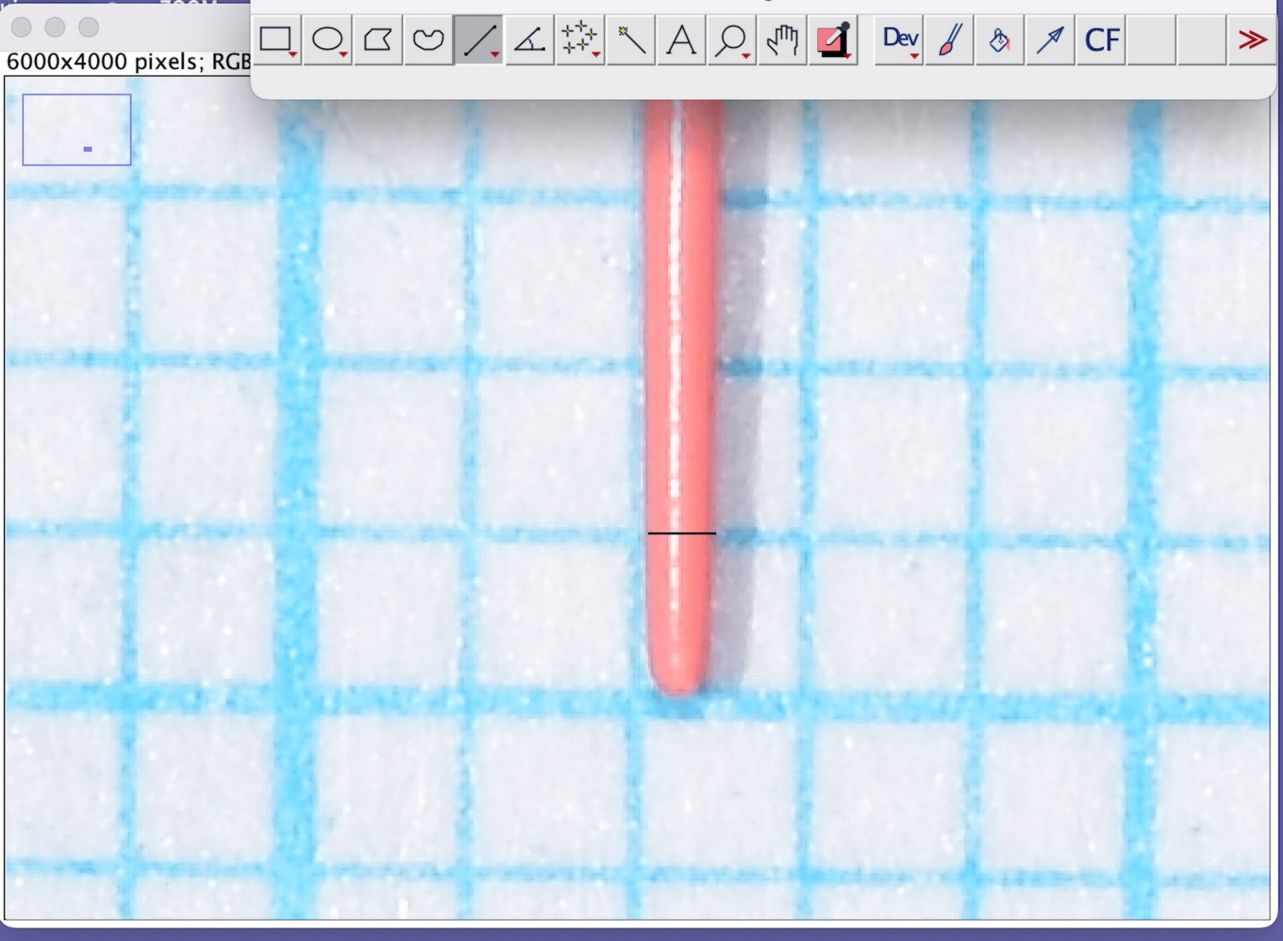
Measurement of a sample in ImageJ software Gutta-percha fixed on the millimetric paper was photographed, and diameters were measured at 1, 2, and 3 mm distances (D1, D2, D3) from the cone tip using the software.

Images in a JPEG format were imported into ImageJ software (version 1.44a; National Institutes of Health, Bethesda, USA), which was used to measure the diameter of the GP cones (Figure 1). Photographs were captured with an mm scale in the background, and pixel dimensions were calibrated in ImageJ based on known reference distances. A line was drawn along a defined length on the grid, and the actual distance was entered in mm using the "Set Scale" function. This calibration ensured that all subsequent measurements were accurately recorded in mm.

The diameters of the GP cones were measured at 1 mm (D1), 2 mm (D2), and 3 mm (D3) from the cone tip, and the diameter differences between D1-D2 and D2-D3 were calculated. To assess measurement reliability, 10% of the GP cone samples were remeasured, and intraobserver consistency was assessed using the intraclass correlation coefficient (ICC).

Statistical analysis

Data were analyzed using IBM SPSS Statistics, version 30.0 (IBM Corp., Armonk, NY, USA) and Python. Descriptive statistics (mean, median, minimum, maximum, standard deviation) were reported. The Shapiro-Wilk test was used to assess data normality.

For comparisons between independent groups, the independent samples t-test was employed when the data exhibited normal distribution, while the Mann-Whitney U test was used for non-normally distributed data. Differences in diameter increase values at the D2-D1 and D3-D2 levels were compared between the two manufacturers (for Ocean and VDW.ROTATE) and taper sizes (0.04 and 0.06). Intra-examiner reliability was assessed using ICC analysis. A p-value < 0.05 was considered statistically significant.

## Results

The ICC between measurements performed by the same examiner at different time points was 0.9967, indicating an excellent level of agreement. The mean and median (minimum-maximum) diameter values of the GP cones at levels D1, D2, and D3 for both the manufacturers and the taper are shown in Table 1.

**Table 1:** Diameter measurements (D1, D2, and D3) of gutta-percha cones in accordance with the manufacturer and taper The Mann-Whitney U test was used for analysis. p ≤ 0.05 was considered statistically significant.

	Mean ± SD	Median (minimum-maximum)	p-value	Z value
Diameter				
D1				
Ocean 0.04	0.3448 ± 0.013	0.3423 (0.3218-0.3898)	0.054	-0.1930
VDW 0.04	0.3391 ± 0.006	0.3428 (0.323-0.3594)		
D2				
Ocean 0.04	0.3812 ± 0.016	0.3768 (0.3550-0.4263)	0.994	-0.008
VDW 0.04	0.3797 ± 0.009	0.3797 (0.3625-0.4063)		
D3				
Ocean 0.04	0.4198 ± 0.017	0.4193 (0.3797-0.4495)	0.562	-0.580
VDW 0.04	0.4220 ± 0.009	0.4187 (0.4124-0.4509)		
D1				
Ocean 0.06	0.3811 ± 0.015	0.3589 (0.3566-0.44)	<0.001	-8.162
VDW 0.06	0.3589 ± 0.004	0.3602 (0.3504-0.3684)		
D2				
Ocean 0.06	0.4295 ± 0.015	0.4192 (0.3966-0.4832)	<0.001	-4.133
VDW 0.06	0.4192 ± 0.004	0.4185 (0.4092-0.4295)		
D3				
Ocean 0.06	0.4850 ± 0.015	0.4831 (0.456-0.5199)	0.005	-2.838
VDW 0.06	0.4782 ± 0.006	0.4795 (0.4638-0.4894)		

When evaluated in terms of the diameter difference between the two manufacturers at levels D1, D2, and D3, in the 0.04 taper cone group, no significant difference was found between the two manufacturers at any levels (Table 1). However, significant differences were observed in the 0.06 taper cone group; the diameters of the GP cones in the Ocean group were significantly larger than those of VDW.ROTATE at D1 (p < 0.001), D2 (p < 0.001), and D3 (p = 0.005).

The diameter increase values between the D2-D1 and D3-D2 levels are shown in Table 2 with respect to the taper group and manufacturer.

**Table 2:** D3-D2 and D2-D1 measurements according to the manufacturer and taper groups ^†^Independent samples t-test ^‡^Mann-Whitney U test ^*^p < 0.05 p ≤ 0.05 was considered statistically significant.

	Mean ± SD		p
	D2-D1		
Taper	0.04	0.06	
Ocean	0.0364 ± 0.008	0.0484 ± 0.011	<0.001^†,*^
VDW.ROTATE	0.0406 ± 0.008	0.0603 ± 0.007	<0.001^†,*^
p	0.005^†,*^	<0.001^†,*^	
	D3-D2		
Taper	0.04	0.06	
Ocean	0.0385 ± 0.010	0.0555 ± 0.014	<0.001^‡,*^
VDW.ROTATE	0.0423 ± 0.009	0.0590 ± 0.007	<0.001^‡,*^
p	0.053^‡^	0.005^‡,*^	

For both manufacturers, the increase in D2-D1 measurements was significantly greater at the 0.06 taper than at the 0.04 taper (p < 0.001). VDW.ROTATE showed a significantly greater increase in D2-D1 values compared to Ocean at both the 0.04 (p = 0.005) and 0.06 taper levels (p < 0.001), and similarly, D3-D2 values increased significantly at the 0.06 taper compared to the 0.04 taper in both groups (p < 0.001). Although the inter-manufacturer difference at the 0.04 taper was not significant (p = 0.053), VDW.ROTATE exhibited a significantly greater increase in D3-D2 values than Ocean at the 0.06 taper (p = 0.005).

## Discussion

The quality of the apical third of the root canal filling is a critical determinant of the long-term success of endodontic treatment [15,16]. Inadequate obturation of the root canal system remains a major contributor to treatment failure [17], with one of the underlying causes being the lack of standardization and dimensional uniformity in both endodontic instruments and GP cones [18]. The most widely adopted approach for obturating the prepared root canal space involves the use of GP cones in combination with a root canal sealer [19]. These cones are manufactured in standard or nonstandard forms, featuring various tip sizes and tapers. Although international standards have been established by organizations such as the American National Standards Institute and ISO to regulate the dimensions of GP cones [7], inconsistencies in their production persist [20]. This lack of conformity poses a significant challenge in clinical practice, potentially compromising the quality of the apical seal and the overall success of root canal therapy [8]. This study aimed to evaluate the apical dimension variability of ISO size 30 gutta-percha cones with 0.04 and 0.06 tapers from two different manufacturers and to assess the impact of taper- and manufacturer-related differences on the standardization.

Numerous studies have investigated the compatibility of GP cones with various file systems [6,7,11,12]. However, the majority of research on the dimensional variability of GP cones has focused primarily on standardized cones with a 0.02 taper [21,22], as well as those with a 0.04 taper [10,23]. To the best of our knowledge, the present study is the first to evaluate the diameter and taper variability at the D1, D2, and D3 levels in ISO size 30 GP cones with 0.04 and 0.06 tapers, obtained from two different commercial sources.

In this study, the diameters of GP cones from two manufacturers (i.e., VDW.ROTATE and Ocean) at points D1, D2, and D3 and the diameter increases between levels D1-D2 and D2-D3 were measured, and the differences were compared. In the 0.04 taper cone group, no significant difference in the GP cone size was observed between the two manufacturers. In contrast, within the 0.06 taper group, the diameters of the Ocean GP cones were significantly larger than those of VDW at D1, D2, and D3. Both manufacturers exhibited greater increases in D2-D1 and D3-D2 values at the 0.06 taper compared to the 0.04 taper (p < 0.001). Furthermore, VDW.ROTATE showed a significantly greater increase in D2-D1 values than Ocean at both tapers (p = 0.005 and p < 0.001) and a higher D3-D2 increase at the 0.06 taper (p = 0.005).

The diameter tolerance is specified for 0.02 taper cones, but no taper tolerance is included in the specification for 0.04 and 0.06 taper cones. In addition to the absence of strict specification tolerances, the considerable dimensional variability of GP cones is largely attributed to their high plasticity [24], which allows for deformation due to temperature fluctuations during storage and transportation, even when the manufacturing process is standardized [25].

Inconsistencies in the standardization of GP points may prolong the root canal filling process and contribute to treatment failures, either by leaving the canal incompletely filled or by extending the cone beyond the apical foramen [21]. Changes in the cone tip size during obturation may lead to clinical difficulties and failures [21]. Moreover, the use of master GP cones that do not properly fit is a known factor that contributes to material extrusion [1].

Previous studies have highlighted notable dimensional variability among GP cones produced by different commercial sources. In a study comparing ISO size 30 GP cones with a 2% taper from two South Korean brands, Meta Biomed and DiaDent, cones from Meta Biomed were found to exhibit significantly greater conformity to ISO standards at the apical diameter level [26]. However, no statistically significant differences were observed between the two brands in terms of the overall percentage of correctly and incorrectly sized cones for ISO sizes 35 and 40. Another investigation assessed the tip diameters (D0) of GP cones from various systems, including ProTaper Universal (Dentsply, Ballaigues, Switzerland), Mtwo (VDW), WaveOne (Dentsply), and Reciproc (VDW), and reported a general lack of standardization, with the exception of the R40 size in the Reciproc system [27]. Similarly, a study examining cones from three different manufacturers, Tanari Man (Manacapuru, Brazil), Cone Tech (Manaus, Brazil), and Dentsply (Tulsa, USA) found that Cone Tech produced the lowest percentage of inadequately sized cones for both 0.04 and 0.06 taper points, with rates of 52% and 50%, respectively [28]. These findings indicate that dimensional accuracy can vary substantially depending on the specific production batch, manufacturing process, and quality control measures employed by each brand [10].

A study evaluating five different ISO size 30 GP cones with a 0.04 taper reported significant variability in both tip diameter (D0) and taper among the brands examined, highlighting the inconsistency in dimensional standards across different manufacturers [10]. Such lack of standardization may lead to increased reliance on the sealer to compensate for discrepancies, potentially prolonging the obturation process and affecting the quality of the root canal filling [29].

According to Bajaj et al. [7], an experienced endodontist can manage the diameter and tip size variability of a master cone. However, for less-experienced clinicians, this type of disparity can be a time-wasting headache. Before root canal obturation, it is necessary to radiographically check the fit of the main cone to the canal. If it is shorter than the working length, the appropriate choice of GP should be reconsidered [20].

Scanning microscopy studies have also shown that GP tips from different manufacturers have artifacts such as large protrusions or deep cratered areas containing numerous free or trapped crystal-like particles [22]. It may be necessary and important to use bioceramic-based root canal sealers and thermoplasticized techniques to prevent inadequate GP fitting in the apical third or to correct structural defects in the GP [30].

As in the present study, 0.06 taper GP may lock coronally due to the lack of standardization, and in this case, there is a potential to perform a canal filling shorter than the working length [21]. Experienced clinicians can detect premature locking of the main GP cone and resolve this issue. To reduce variability, standardization organizations may consider narrowing the diameter and taper tolerance ranges for all endodontic files and GP cones.

Strengths and limitations

The strengths of this study include the use of digital image analysis, which provided precise and reproducible measurements supported by a high intraobserver reliability (ICC = 0.9967). To minimize selection bias, samples were chosen from different lots and stored under controlled conditions.

Our study was not without limitations. We did not conduct a diameter comparison of hand and rotary files with the corresponding GP points but evaluated the standardization between different manufacturers. The coronal and middle thirds were not evaluated, and only the diameters in the apical third were measured. In this study, the first measured diameter was D1, not D0. According to the limitations of the studies present in the literature, it was not possible to reliably measure D0 because of the rounded, non-cutting tip design of the file specimens [6,11,12]. ​​The effects of canal anatomy and preparation on the cone fit were not evaluated. Further research, including both in vitro and clinical studies, is needed to determine whether GP diameter discrepancies, such as those in this study, affect the clinical use of GP cones.

## Conclusions

In conclusion, this study found that there is dimensional variability among gutta-percha cones from different manufacturers, despite identical taper specifications. Such inconsistencies may adversely affect cone adaptation and sealing ability during root canal obturation, increasing the risk of reinfection and potentially leading to overextended GP and subsequent postoperative pain. Particular attention should be given to cones with greater taper values, where deviations appear more pronounced. These findings emphasize the importance of verifying the cone fit during clinical procedures and highlight the need for enhanced manufacturing precision to improve standardization and ensure more consistent endodontic outcomes.
